# On the potential of transfer entropy in turbulent dynamical systems

**DOI:** 10.1038/s41598-023-49747-1

**Published:** 2023-12-15

**Authors:** Daniele Massaro, Saleh Rezaeiravesh, Philipp Schlatter

**Affiliations:** 1 SimEx/FLOW, Engineering Mechanics, KTH Royal Institute of Technology, Stockholm, 100 44 Sweden; 2 Department of Fluids and Environment/MACE, The University of Manchester, Manchester, M139PL UK; 3 Institute of Fluid Mechanics (LSTM), Friedrich-Alexander-Universität (FAU) Erlangen-Nürnberg, Erlangen, 91058 Germany

**Keywords:** Fluid dynamics, Information theory and computation

## Abstract

Information theory (IT) provides tools to estimate causality between events, in various scientific domains. Here, we explore the potential of IT-based causality estimation in turbulent (i.e. chaotic) dynamical systems and investigate the impact of various hyperparameters on the outcomes. The influence of Markovian orders, i.e. the time lags, on the computation of the transfer entropy (TE) has been mostly overlooked in the literature. We show that the history effect remarkably affects the TE estimation, especially for turbulent signals. In a turbulent channel flow, we compare the TE with standard measures such as auto- and cross-correlation, showing that the TE has a dominant direction, i.e. from the walls towards the core of the flow. In addition, we found that, in generic low-order vector auto-regressive models (VAR), the causality time scale is determined from the order of the VAR, rather than the integral time scale. Eventually, we propose a novel application of TE as a sensitivity measure for controlling computational errors in numerical simulations with adaptive mesh refinement. The introduced indicator is fully data-driven, no solution of adjoint equations is required, with an improved convergence to the accurate function of interest. In summary, we demonstrate the potential of TE for turbulence, where other measures may only provide partial information.

## Introduction

Galileo Galilei based the scientific method on building hypotheses, making predictions and then collecting empirical observations founded on those predictions^1^. With such an ongoing cyclic structure, the capability of making predictions relies on the possibility of pointing out causal relationships between different observations. The assessment of causal inference gets more challenging as the system’s complexity increases. However, in the last decades, new mathematical and computational tools have become available. Among those, the Information Theory (IT) discipline allows quantifying to which extent each variable of the system contributes to information production and the rate of exchanging information among each other^2^.

Information theory describes the governing laws of information, i.e. the science of message communication. A message is defined as encoded information, such as the bits used to encapsulate text or the nerve signals that cause our lungs to contract. The amount of information in a message generated by a system can be quantified by the Shannon entropy^3^. Similarly to the thermodynamic entropy^4^, it constitutes a measure of the state of disorder and uncertainty. Shannon was mainly interested in estimating the amount of information transmitted in a communication system, but IT metrics enable the establishment of the cause-effect relationships among processes of a generic system. This is due to the link between the information flux and the one-way direction of time, also known as time asymmetry^5^. Since all the laws of physics are time-symmetric at the microscopic level and hence reversible^6^, in principle, the arrow of time is settled by the asymmetry which arises macroscopically in the system. IT introduces some metrics based on the Shannon entropy to statistically measure these asymmetries, see also Refs.^7,8^. Among those, one non-intrusive possibility is referred to Wiener^9^, who introduced transfer entropy (TE). This shares some of the mutual information properties but takes the dynamics of information transport into account. Albeit Schreiber^2^ formally derived the transfer entropy (see also Bossomaier et al.^10^), its prediction introduces many parameters which potentially affect the results in a significant manner. These include the time lag between the source and target signals. As a result, the assessment of transfer entropy is far from well-established, especially in complex dynamical systems^11,12^.

Inspired by the success of IT in various disciplines^13,14^, we decide to discuss and assess the transfer entropy to use it in chaotic dynamical systems (DS). The most famous example of a chaotic DS is probably the Lorenz system^15^. This is a simplified mathematical model for atmospheric convection, which describes a fluid in two-dimensional (2D) motion, initially at rest, uniformly warmed from the bottom and cooled from the top, see Eq. (1). It predicts the air convection and temperature variations in the atmospheric boundary layer. The model equations describe the evolution in time of the quantities *x*, proportional to the rate of convection, *y* and *z* proportional to the horizontal and vertical temperature variation, respectively. The constants $\sigma$, $\alpha$ and $\beta$ are parameters that represent the Prandtl number, Rayleigh number, and the layer’s length scale, respectively. In the current example, we consider the typical $\sigma =10$, $\alpha =28$ and $\beta =-8/3$:

1 $$\begin{aligned} \begin{aligned} \frac{dx}{dt}&= \sigma (y-x) \\ \frac{dy}{dt}&= x(\alpha -z) -y \\ \frac{dz}{dt}&= xy -\beta z, \end{aligned} \end{aligned}$$

**Figure 1:**
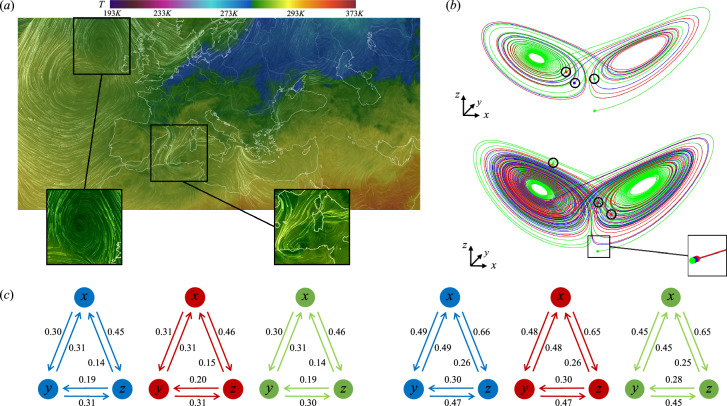
(**a**) Lagrangian wind patterns (white) in the atmosphere over central Europe and the eastern Atlantic Ocean on January, 25th 2023. The temperature contour plots are at the altitude of $1000\, hPa$. Distinguished trajectories, in the selected squares, qualitatively resemble Lagrangian coherent structures in the atmosphere^17^. The TE can potentially unveil the causal relations within such mechanisms as shown below for the Lorenz system. The map is adapted from the open source Ref.^18^. (**b**) The Lorenz attractor in the phase space shows the exponential divergence of the solution starting from three similar but slightly perturbed initial conditions: $(x=1+\epsilon ,y=1+\epsilon ,z=1+\epsilon )$ with $\epsilon \approx 10^{-4}$, after $t_1=25$ (top) and $t_2=100$ (bottom) time units, respectively. The final states are highlighted by black circles. In (**c**), the transfer entropy is computed among the time series of *x*, *y* and *z* (proportional to the rate of convection, the horizontal and vertical temperature variations, respectively) at $t_1$ (left) and $t_2$ (right), respectively.

As shown in Fig. 1b, we consider three initial conditions slightly perturbed $(x=1+\epsilon ,y=1+\epsilon ,z=1+\epsilon )$ with $\epsilon \approx 10^{-4}$. After some time, the well-known butterfly attractor^16^ is shown in Fig. 1. While the three trajectories exponentially diverge in phase space, the transfer entropy computed at two different times does not show significant variations in the three trajectories and provides a clear indication of the causal relations among *x*, *y* and *z*. In Fig.  1c, on the one hand, the rate of convection *x* and the horizontal temperature *y* are equally causing each other. On the other hand, the rate of convection *x* is the main responsible for the changes in the vertical temperature variation *z*. At the same time, *z* is mostly caused by *y*. The Lorenz system is a simple but instructive example of how to exploit transfer entropy to find and quantify the causality in a chaotic system. Similar attractors can be found in natural systems, e.g. the atmosphere and the ocean, and identified by Lagrangian coherent structures (LCS). These structures play a critical role because they help identify regions in a fluid flow where particles tend to stick together or separate, providing insight into the overall flow behaviour. The LCS can predict the movement of pollutants, debris, or the dispersion of organisms (see Fig. 1a). This represents a further application where TE can unveil the causality behind such complex mechanisms.

Given that, our initial focus lies in examining the impact of different hyperparameters in a low-order vector auto-regressive model (VAR), i.e. a set of autoregressive models (ARMs) which are cross-covariated. In this pursuit, we introduce the transfer entropy function (TEF), drawing an analogy to the auto-correlation function (ACF). Note that correlation (cross and auto) is the associated covariance normalised by the variance of the time series^19^. Next, we shift our focus to a more complex chaotic system, namely, the incompressible Navier–Stokes (NS) equations in the turbulent regime, which serve as a realistic model for various flows. The transfer entropy exhibits numerous strengths, being a non-intrusive approach that only necessitates at least two time series to analyse. Additionally, our study highlights the connections between the TE and other extensively used tools in turbulence research, e.g. the cross-covariance. Eventually, related to the direct numerical simulations of the incompressible NS equations, we exploit the IT metrics to propose novel machinery for controlling numerical errors, where the TE serves as a sensitivity indicator for automatic grid refinement in computational fluid dynamics (CFD). This novel application can be of interest for CFD developments targeting exascale simulations^20^.

## Results

### Low-order vector autoregressive model

To assess the TE, several hyper- and numerical parameters are required to be defined. The degree to which the TE estimation is impacted by them has yet to be determined. To begin tackling this issue, we consider samples generated from a first-order vector autoregressive model^21^ defined as:

2 $$\begin{aligned} \begin{bmatrix} x_i \\ y_i \end{bmatrix} = \begin{bmatrix} a_0 \\ b_0 \end{bmatrix} + \begin{bmatrix} a_1 &{} a_2 \\ b_1 &{} b_2 \\ \end{bmatrix} \begin{bmatrix} x_{i-1} \\ y_{i-1} \end{bmatrix} + \begin{bmatrix} \epsilon _{x_i} \\ \epsilon _{y_i} \end{bmatrix}, \end{aligned}$$

where the associated noise samples are chosen to be Gaussian and correlated:

3 $$\begin{aligned} {[}\epsilon _x,\epsilon _y]^T \sim {\mathcal {N}}({\textbf{0}},{\textbf{C}}_\epsilon ),\quad {\textbf{C}}_\epsilon = \begin{bmatrix} a_3^2 &{} \rho _\epsilon a_3 b_3 \\ \rho _\epsilon a_3 b_3 &{} b_3^2 \end{bmatrix}. \end{aligned}$$

**Figure 2:**
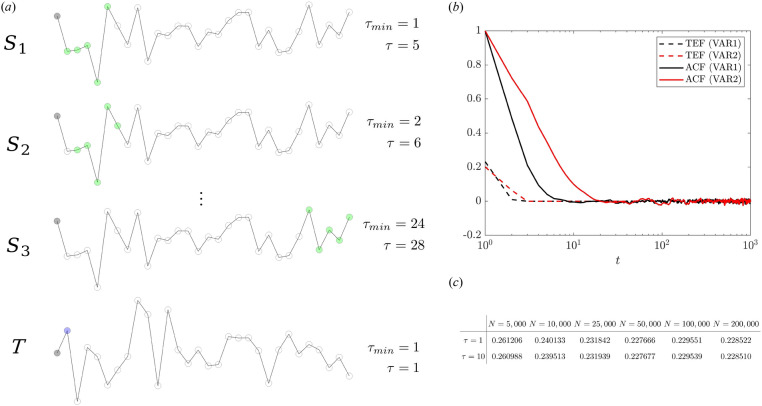
(**a**) Illustration of the TE estimation given two time series, i.e. the source *S* and target *T*, with samples drawn from a bivariate Gaussian distribution. For both *S* and *T*, several parameters must be defined: *i*. the maximum time lag $\tau$, i.e. the Markovian order, *ii*. the minimum time lag  $\tau _{min}$ that indicates a time shift for the beginning of the region of influence taken into account, and *iii*. the transfer entropy estimation window $L_w=\tau -\tau _{min}+1$. For the target, we always consider $\tau _{min}=1$ and $\tau =1$. For the time signals *S* and *T*, each bullet corresponds to a sample: the present state is black (marching in time from left to right), the past states for the source *S* and the target *T* are in green and in blue, respectively. Considering a Markovian order for the source equal to 5 ($\tau =5$) and no time shift ($\tau _{min}=1$), the entropy transferred to the present target state is computed. The time shift (*t*) introduced in the transfer entropy function (TCF) calculation, in analogy with the autocorrelation function (ACF), corresponds to $\tau _{min}$. The considered green *S* states are progressively shifted and $L_w$ is kept constant. The examples for $t=1$ ($S_1$), $t=2$ ($S_2$) and $t=24$ ($S_3$) are shown. (**b**) The TCF and ACF for the first and second-order vector autoregressive models as a function of the *t* time shift. (**c**) The transfer entropy between two time series generated from the first-order vector autoregressive model (2) for a progressively larger number of samples *N*. Two different Markovian orders are considered: $\tau =1$ and $\tau =10$.

The time series *x* and *y* are the source *S* and target *T*, respectively. We ensure stationary conditions, i.e. the model parameters $a_i$, $b_i$ and $\rho _{\epsilon }$ are chosen to have the eigenvalues of the square matrix in (2) lying within the unit circle. Including  $x_{i-2}$ and $y_{i-2}$, the model (2) has also been extended for a second-order VAR. In both scenarios, we first draw initial samples for *x* and *y* from ${\mathcal {U}} \in [0,1]^2$. Then the model is used to generate 2*N* samples. The initial *N* samples are discarded to account for the burn-in process and obtain a statistically stationary time series with *N* samples^22^. The number of samples *N* can potentially constitute a source of uncertainty in the entropy estimation. Nonetheless, previous studies^23^ have shown that the Shannon transfer entropy estimation is robust and works well even for relatively small sample size. Our results confirm past observations^23^: when *N* is larger enough to capture the relevant dynamics (e.g. $N\ge 50,000$ in the current case) no difference is observed in the TE up to the second digit, see the table in Fig. 2c. A similar trend is observed for $p=2$ (not shown here). Furthermore, we extend the previous analysis^23^ by also looking at different Markovian orders $\tau$, i.e. the maximum time lags considered when computing the TE. In Fig. 2c, $\tau$ is chosen to be the same for both source and target. For the first-order VAR, the table shows that there is no distinction between considering the first and tenth Markovian orders. The result remains unchanged for the second-order VAR. Therefore, the TE calculation is not affected by taking a time lag, i.e. a Markovian order, larger than *p*, where *p* is the order of the VAR. Contrary, the computational cost is largely affected by choosing a maximum time lag ten times larger, as discussed below. Unlike the maximum time lag, which corresponds to the Markovian order, the minimum time lag $\tau _{min}$ indicates a time shift in the past which allows excluding the influence of a certain interval on the current state. A clarification of the meaning of $\tau _{min}$ is provided in Fig. 2a. In contrast to previous works, where a variable time lag is considered^24^, we introduce the transfer entropy function (TEF) in analogy with the autocorrelation function (ACF). When computing the ACF, we estimate for how long in time, or for how many time steps, a process is correlated with itself (at the current state)^25^. Similarly, the TEF measures how far in the past the source is causally related to the current state of the target (also, knowing how much about the past of the source would lead to the reduction of uncertainty in the future of the target). As an example, given $\tau _{min}=1$ and $\tau =5$ for the source ($\tau _{min}=1$ and $\tau =1$ for the target), we consider the causality generated by the last five samples on the current state of the target with a time window for the TE estimation equal to $L_w =5$. In the TEF estimation, when the time shift *t* (corresponding to $\tau _{min}$ as explained in Fig. 2) increases to $\tau _{min}=2$, by keeping a fixed $L_w$, we consider the entropy transferred between the source interval [2, 6] (instead of [1, 5]) and the current target state. The TEF in Fig. 2b drops to zero for $t=1$ and $t=2$ for the first- and second-order VAR, respectively. The result shows that the VAR order determines the system embedding time for the transfer entropy. Differently, for the ACF the system embedding time is defined by the integral time-scale of the time series^26^.

### The turbulent channel flow

To comprehensively assess TE as a causality measure, we apply it to complex dynamical systems, specifically turbulent flows. These flows are ubiquitous and profoundly influence multiple facets of our daily lives, also appearing in many engineering applications. In recent decades, the fluid dynamics community has been actively exploring a multitude of tools to gain new physical insights. Previously, different kind of causality measures has been used to study the energy redistribution among turbulence scales^28–32^, the interaction of porous media with free turbulent flow^33^ and more recently, for subgrid-scale modelling in large eddy simulation (LES)^8^. Conversely, in the present work, we begin with the analysis of the TE variation with the wall-normal distance since the near wall dynamics, with its self-sustaining cycle, constitutes the root cause of friction drag^34^. Then, we discuss the relation between the transfer entropy and other standard statistical tools, e.g. the cross-covariance. As we focus on wall-bounded turbulence, it is necessary to introduce the viscous length $\delta _{\nu } = \nu /u_{\tau }$, the friction velocity $u_{\tau }=\sqrt{\tau _w/{{\bar{\rho }}}}$, where $\tau _w$ is the wall shear stress and ${{\bar{\rho }}}$ is the density, in contrast to $\rho$ indicating the correlation coefficient. The friction-based Reynolds number is $Re_{\tau } = u_{\tau } \delta /\nu$, here $\nu$ is the kinematic viscosity and the outer layer characteristic length is taken to be $\delta =h$, the channel half-height (here $Re_{\tau }=300$). The wall distance is also expressed in viscous length units as $y^+=y/\delta _{\nu }$.

**Figure 3:**
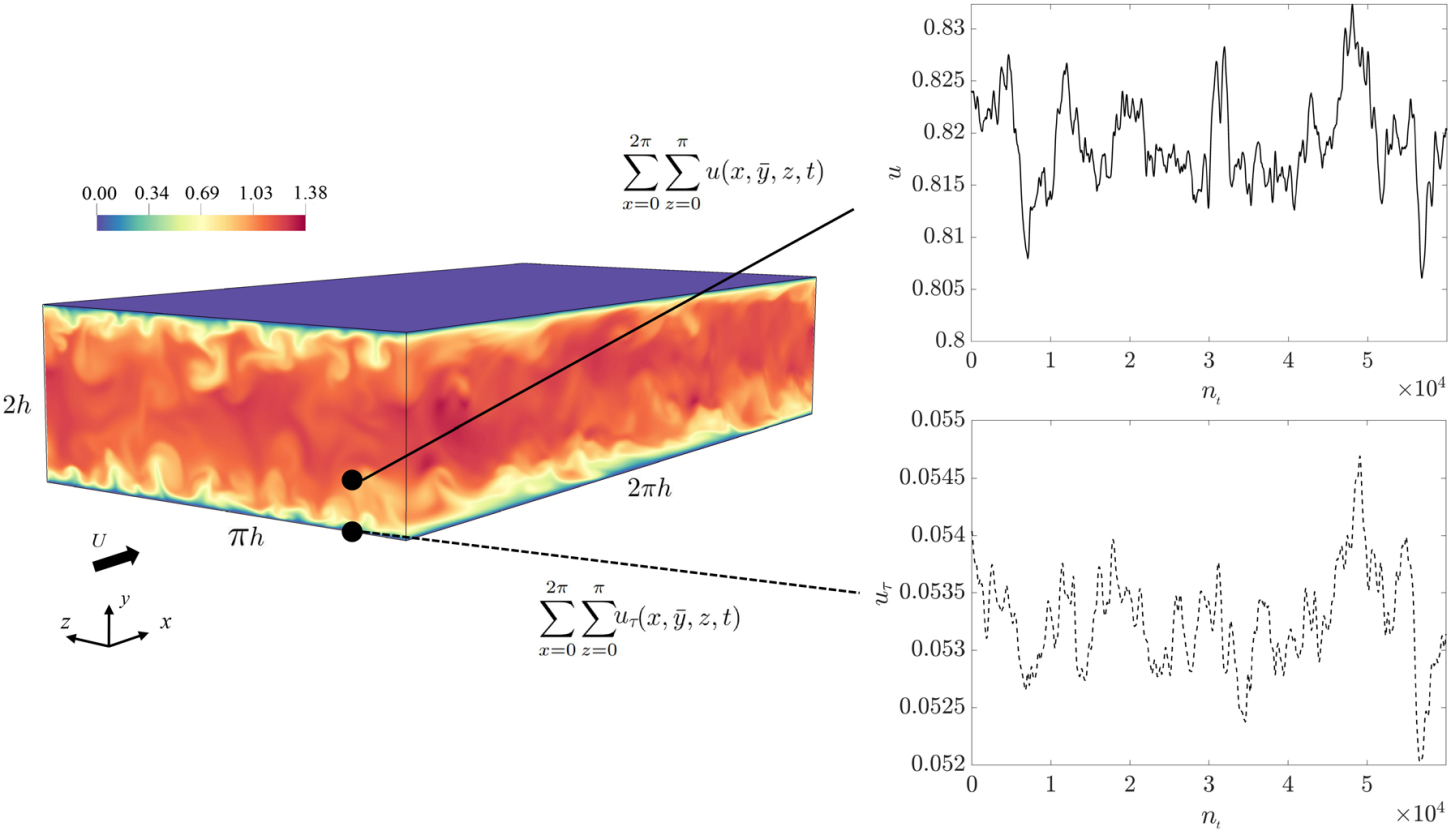
Sketch of the computational setup: Representation of an instantaneous velocity magnitude field of the turbulent flow at $Re_{\tau }=300$ and two time series (in relation to the number of time samples ($n_t$), spatially averaged in the streamwise and spanwise directions) extracted for the friction and the streamwise velocities $u_\tau$ and *u*, respectively.

The standard observable “causal” relation in the investigation of turbulence has been the correlation in time. But what does the correlation really tell us? Is it indeed a causality measure? The answer is: not always; and even when it is, it does not tell everything. In the given channel flow (Fig. 3), let us consider a fixed distance from the wall $y^+=30$, where the Pearson, or linear, correlation coefficient between the streamwise velocity and the friction velocity (both spatially averaged in the streamiwse and spanwise directions) is $\rho _{u-u_{\tau }}=\rho _{u_{\tau }-u}=0.54$. Given that $\rho$ varies between $-1$ and 1; the coefficient indicates a relatively high level of linear correlation, as one could expect. However, the question remains: what is the cause? The time correlation can not answer as it is a symmetric measure, but the transfer entropy estimation shows a clear causal direction: ${TE}_{u-u_{\tau }}=0.0448$ (*u* is the source and $u_{\tau }$ is the target) and ${TE}_{u_{\tau }-u}=0.353$. It is worth noting that, according to the TE definition in Sect. Causality as a measure, the transfer entropy can vary between 0 and 1. At $y^+=30$, the net information goes from $u_{\tau }$ to *u*, i.e from the wall to the flow. We thus clearly observe that knowing the history of $u_{\tau }$ improves our understanding of *u* and not vice versa ($u_{\tau }$ is mainly causing *u*). The role of the wall as an information source is a remarkable finding. The dominant directionality of the TE from the wall to the flow away from the wall is a crucial observation that can help tackle challenging tasks, such as the development of novel techniques for drag reduction (DR), e.g. by moving walls^35,36,37^. For this family of DR techniques, such a causality of the wall boundary condition to the outer region is pivotal, as it ensures that wall control can significantly influence flow further from the wall. Previous approaches failed in measuring such causality by using cross-correlation. Differently, the TE depicts the directional information in a clear way. At this point, it is interesting to explore the extent to which the wall actively influences the streamwise velocity. This also results in a deeper comprehension of the interplay between the wall and the near-wall velocity streaks. Indeed, the TE varies in a non-trivial way with the wall distance, as described below.

**Figure 4:**
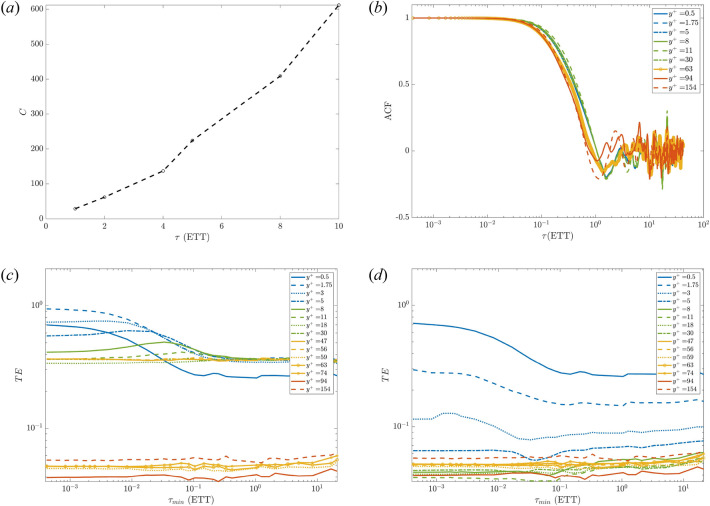
(**a**) Estimation of the cost (*C* expressed in seconds) considering a progressively larger history of the source ($\tau$). The calculation involves just two time series: the streamwise velocity and the wall friction velocity as source and target, respectively. The minimum time lags $\tau _{min}$ are set to one. (**b**) The auto-correlation function of the velocity *u* at different wall-normal distances as a function of the time delay expressed in terms of eddy-turnover-time. The bottom panel represents the TE between the wall friction velocity $u_{\tau }$ and streamwise velocity *u* at various wall normal distances. In (**c**), $u_\tau$ acts as the source with the streamwise velocity being the target. This order is reversed in (**d**). A longer history effect is taken into account by using $\tau =10$. The velocity probes located in the viscous sublayer ($y^+<5$), in the buffer layer ($5<y^+<30$), in the log-law region ($30<y^+<0.3y/h$, for $Re_\tau =300$ this reads $30<y^+<90$) and in the outer region ($y^+>90$) are coloured in blue, green, orange and red, respectively. The layers classification follows Pope^27^, noting the comparably short extent of the logarithmic region due to the low Reynolds number.

Thus, we look at the TE variation away from the wall. Figure 4c,d show how TE varies with $\tau _{min}$ at different locations. Here, $\tau _{min}$ is expressed in eddy turnover time (ETT) which is defined as ETT$=u_{\tau }/h$ and characterises an integral time of turbulence associated with the lifetime of larger vortices. This represents an estimation of the time scale required to transfer energy from the large to the small scales. We report that the wall-frictional velocity (*source*) is causing, with a net transferred entropy, the streamwise velocity (*target*) for $y^+ \le 56$. Conversely, for $y^+\ge 59$ no significant information is transmitted based on the negligible values of the TEs. This is clear in Fig. 4c, where the TE drops below 0.05, i.e. less than 5% of the level of information for the target arises from the source. Hence, we consider the level of causation of $u_{\tau }$ not significant since other sources contribute for the remaining 95%. Furthermore, the TE shows remarkable differences compared to the auto-correlation function which has been widely used in the past for turbulent channel flows^38,39^. First, the TE does not level out to zero, rather it reaches an asymptotic value larger than zero, around 0.5 ETT (at least for a limited time interval $\approx 40$ ETT). Moreover, the TE drops down after a longer time, whereas the ACF dropping is more abrupt (both around $1 \sim 5$ ETT), see Fig. 4b. It is also worth noting the significantly different computational costs of computing TE and ACF. On the one hand, the ACF has a negligible cost of the order of a few seconds. On the other one, the cost (*C*) of estimating TE scales almost linearly with the number of history time lags taken from the source, see Fig. 4a. The cost scaling was performed in serial on a workstation equipped with an Intel i9 CPU (14 cores).

Eventually, we observe the connection between the TE and the cross-covariance in a such high-dimensional chaotic dynamical system. The cross-covariance measures the degree of similarity between two processes: *x* and the lagged *y*, as a function of the time lag *k* (with both signals always demeaned). It determines to what extent they match up and, particularly, at what lag the best match occurs: $\gamma _{xy}(k)=\text {Cov}(x_{i}, y_{i+k})$ is the cross-covariance function (CCovF). If normalised, the cross-correlation function (CCF) is obtained: $\rho _{xy}(k) = \gamma _{xy}(k)/\sqrt{\sigma ^2_{x_i}\sigma ^2_{y_{i}}}$, where $\gamma _{xy}(k)=\text {Cov}(x_{i}, y_{i+k})$ is the cross-covariance function and $\sigma ^2_{x_i}$ is the variance of *x* at time *i*^19^. The processes *x* and *y* correspond to the streamwise and frictional velocities, respectively. For $k>0$ and $k<0$, we compute $\gamma _{xy}(k>0)$ and $\gamma _{xy}(k<0)$, with the only symmetry $\gamma _{xy}(k)=\gamma _{yx}(-k)$. Note that, the CCovF and TE are both asymmetric metrics considering the directionality of linear correlation and information transfer, respectively.

**Figure 5:**
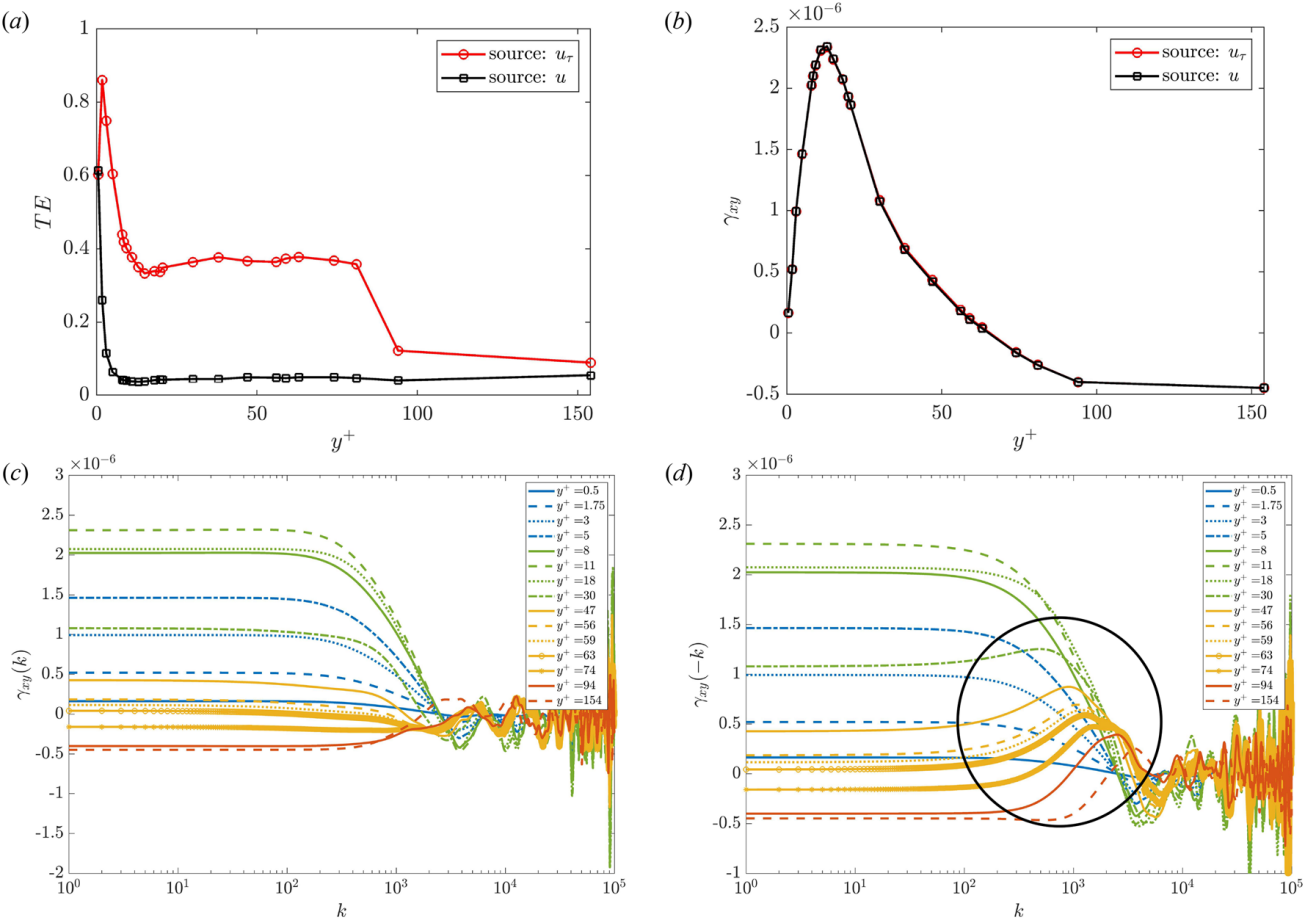
(**a**) Variation of the TE and (**b**) cross-covariance between the source and target with the vertical wall distance. In the TE estimation, the time lag parameters are chosen according to the previous discussion, see Fig. 4. Particularly, $\tau _{min}=1$ and $\tau =10$, where the maximum time lag guarantees that the TE gets an asymptotic value. Analogously, to ensure that the same part of the source’s history is taken into account for calculating the cross-covariance function (CCovF), the same lead/lag is used ($k=10$). (**c**) The CCovF for $k>0$ and (**d**) for $k<0$ at selected $y^+$ locations. The streamwise and wall-friction velocities are denoted by *x* and *y* processes, respectively. The black circle highlights the bump present in $\gamma _{xy}(k<0)$ starting from $y^+>20$, exactly where the TE’s plateau begins.

By looking at the entropy transferred from *u* to $u_{\tau }$ a negligible value (according to the 5% minimum threshold of TE) is reported for $y^+\ge 5$. Instead, from $u_{\tau }$ to *u*, we see that the TE levels out at $y^+=20$, showing its first plateau. Similarly, the CCovF has a peak around $y^+=20$, when it starts to decrease (Fig. 5a,b). Furthermore, for $\gamma _{xy}(k<0)$, which is equal to $\gamma _{yx}(k>0)$, indicates an effect of the past of the $u_\tau$ on the streamwise velocity *u*, evidenced by a bump in the curve for $y^+>20$, corresponding to the beginning of the TE’s plateau. Eventually, both TE and $\gamma _{xy}$ drop to constant values around $y^+\approx 100$. While the source and target show a negative cross-covariance (Fig. 5b), the transfer entropy becomes negligible indicating a weak information transfer (Fig. 5a). Although a concrete explanation of the physics behind these observations is beyond the scope of the present study, a general point to note is that the phase shift between the source and target time series that is related to the turbulence structures and their convection has led to these observations. At the current Reynolds number ($Re_\tau =300$), $y^+\approx 100$ corresponds to the end of the log-law region. It is notable that both metrics highlight a drop in the causality/correlation with this distance from the wall. To the authors’ knowledge, the TE and cross-covariance $\gamma _{xy}$ connection for the turbulent time signals is highlighted here for the first time. The physical interpretation of the bump is not straightforward since the time series are averaged in the spanwise direction. Nonetheless, these trends were confirmed by further tests with pointwise (not averaged in space) time series, indicating turbulent events like *bursts* or similar. However, this is out of the scope of the current work which aims to show the connection between TE and CCovF and to promote the TE as an integral tool to investigate high-order dynamical systems as it is more informative than the CCovF. Indeed, the CCovF suffers from significant spurious oscillations for very high *k*. In addition, it just allows for *k*-point measure, where an accumulation of the past is considered, differently from the TE, which enables the encapsulation of a shift in the history effect, i.e. multiple *k*.

### Causality-based algorithms for computational fluid dynamics

In addition to data analysis of turbulent flows, the causal metrics have a great potential to improve available numerical techniques for turbulence simulation. In particular, we propose here a novel application of IT metrics in CFD which nowadays aims to push the Reynolds number limit further by making the required computational cost more affordable. In this regard, the adaptive mesh refinement technique (AMR) constitutes a necessary tool for next-generation CFD solvers^40^. Our group has implemented the AMR in the open-source spectral-element-based code Nek5000^41^ and assessed its accuracy and reliability^42–45^. One of the main AMR ingredients is the measurement of the committed error in the numerical computation of the Navier–Stokes equations. We implement the spectral error indicator (SEI) by Mavriplis (1989)^46^, which measures and extrapolates the truncation and quadrature errors. The reader can refer to Ref.^47^ and to Refs.^48–50^ for the technicalities and some applications. The main drawback of this standard approach is that the SEI can not refine according to a given quantity of interest (QoI). Differently, other estimators as the adjoint error estimator (AEE)^42,43^ can improve the mesh according to a user-defined objective function *J*. This becomes even more important in direct numerical simulations of turbulent flows where most of the sensitivity analysis fails^51^.

**Figure 6:**
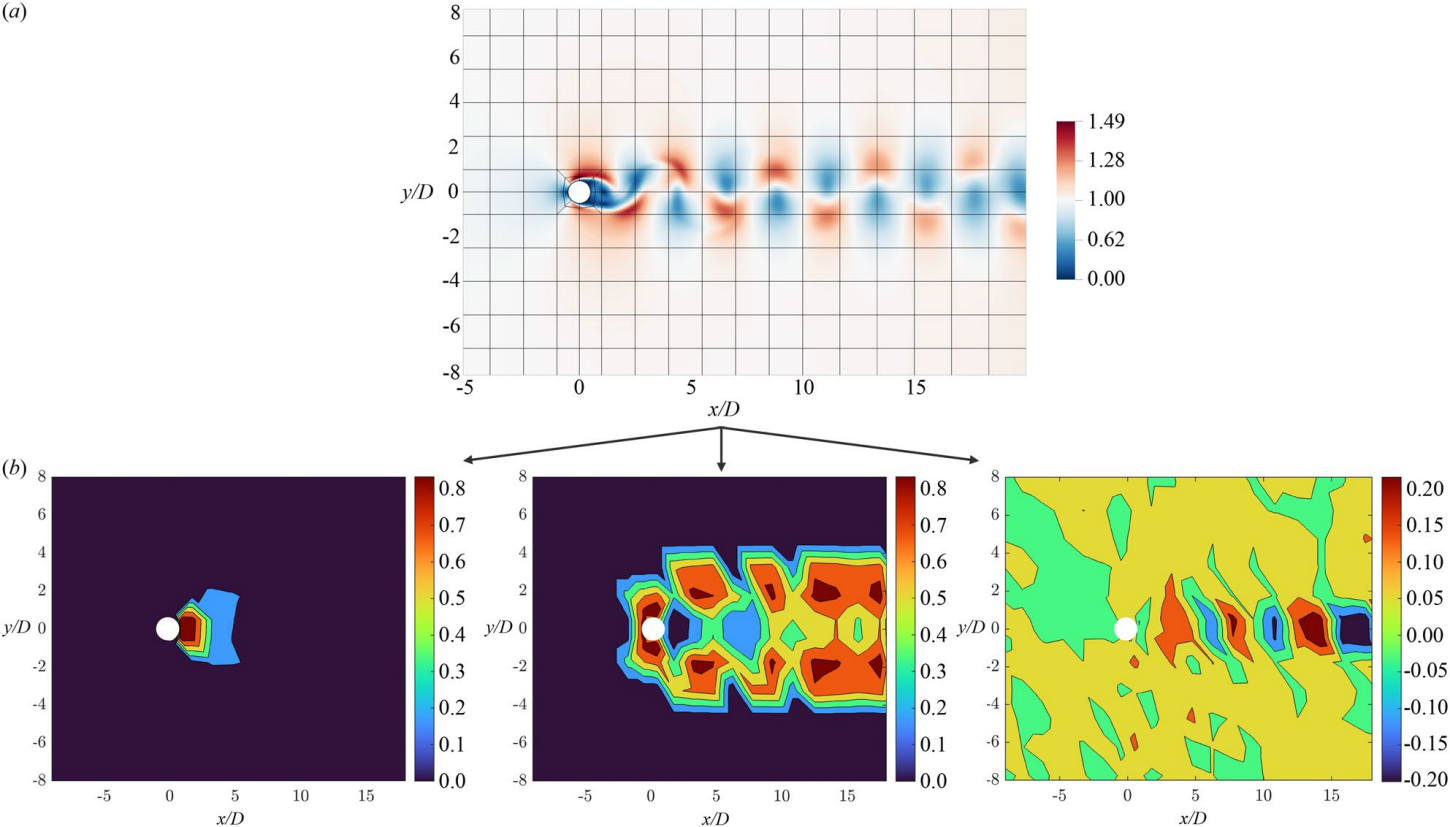
(**a**) The flow around a circular cylinder at $Re_D=200$. The velocity magnitude contour plot and the spectral element grid are shown. Each element contains ($N+1$)$\times$($N+1$) GLL points, where $N=7$ is the polynomial order. (**b**) From left to right: the time-averaged spectral error indicator per element, the transfer entropy and the Pearson correlation coefficient between the velocity magnitude interpolated to the centre of each element (source) and the cylinder drag (target) for the initial mesh. The time series have been collected for at least 10 vortex shedding periods. The SEI varies between 0 and 1 (normalised w.r.t. the maximum), the TE varies between 0 and 1, and the Pearson correlation coefficient can vary between $-1$ and 1.

In the current section, we aim to explore the possibility of using TE as a measure of the causality between different regions of the mesh and *J* for the purpose of driving the grid refinement algorithm. Thus, we can have a method that can automatically refine or coarsen the mesh in the regions of the domain where the solution is, respectively, highly or slightly in causal relation with *J* in a similar way as adjoint-based error estimators are designed^42,43, 52^ for exploiting the sensitivity information. As an example, in the flow around an airfoil or a cylinder, the drag and lift are suitable QoIs. We consider here a circular cylinder, see Fig. 6a. By monitoring the time signals of the velocity field in a given element and the cylinder drag, the TE can be evaluated. Note that the causality measure itself is not sufficient to drive the refinement process as we need a quantity that describes the current mesh quality, i.e. its associated error gets smaller as the mesh quality is enhanced. For this reason, we propose to combine the SEI ($\varepsilon$) with TE introducing a new causality-based error estimator:

4 $$\begin{aligned} \varepsilon _{TE} = \varepsilon \cdot TE_{x \rightarrow y}, \end{aligned}$$

where $TE_{x \rightarrow y}$ is the transfer entropy evaluated for a given time interval *T* from the source *x*, the velocity magnitude interpolated in the centre of each element, to the target *y*, the drag of the cylinder. Therefore, the $\varepsilon _{TE}$ provides a time-averaged error measurement per element. Eventually, the TE acts as a weight for the SEI providing a causality measure w.r.t. *J*, similarly to the adjoint error estimator, where the dual solution provides a local sensitivity^52^.

**Figure 7:**
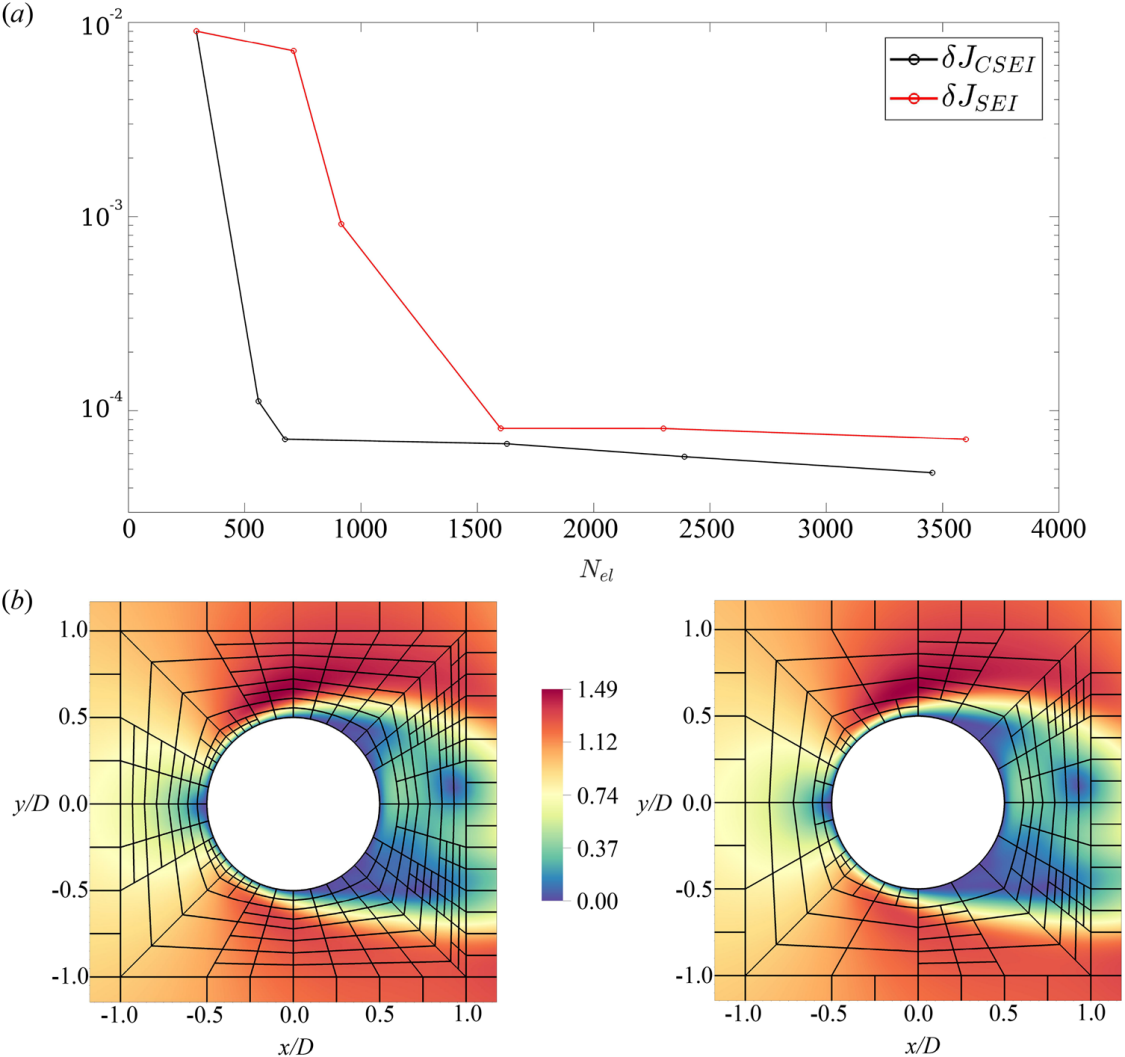
(**a**) The error on the functional of interest, i.e. the drag of the cylinder, with different numbers of spectral elements $N_{el}$. For the SEI and CSEI, the error in the functional of interest $J({\textbf{u}}_{N})$ is measured w.r.t. the reference solution $J({\textbf{u}}_{ref})$: $\delta J=|J({\textbf{u}}_{ref})-J({\textbf{u}}_{N})|$. (**b**) The comparison between the converged mesh obtained through causality-based (*left*) and standard (*right*) spectral error indicators in the flow past a circular cylinder. The element boundaries are shown in black with the velocity magnitude contour plot.

In our simulation, the initial mesh is chosen extremely coarse, with only 292 spectral elements and polynomial order 7 resulting in approximately $1.8e^4$ grid points. The following error indicators are compared: the SEI and the causality-SEI (CSEI). The number of elements is increased by a factor of 10 and the functional *J* is the drag coefficient, $C_D$, on the circular cylinder. The causality between the source $\Vert {\textbf{u}}_i(t) \Vert$ and the target ${C_D}(t)$ is measured for each element *i* with the embedding delay $\tau =10$ for the source and $\tau =1$ for the target (in both terms $\tau _{min}=1$). As previously discussed, the choice of these hyperparameters can play an important role in the causality assessment. In addition, the cost of estimating the transfer entropy in a complex dynamical system can increase significantly by the time lag $\tau$. For the 2D cylinder, the TE estimation is robust when at least one vortex shedding is captured in the source’s history, i.e. $\tau \ge 1$. Here, the total number of samples collected before computing the TE (and performing the refinement) corresponds to 10 vortex-shedding cycles. The SEI is normalised by its maximum global value and the symmetry of the plots in Fig. 6b) is a qualitative indicator of the temporal convergence of the measurement. In Fig.  6b), *centre*, it is clear that the CSEI tends to further refine the region in front of the cylinder, around the stagnation point and in the boundary layer separation locations (Fig. 7b). The CSEI results are in agreement with the adjoint error estimator described by Refs.^42,43^, showing that the TE can potentially provide an alternative to the standard adjoint-based sensitivity analysis techniques while being completely data-driven without the need to solve adjoint equations. For the sake of completeness, the Pearson correlation coefficients are also reported in Fig. 6b, *right*. Regions in the wake, corresponding to the vortex alternations show mildly positive and negative correlations. Nonetheless, around the cylinder, the correlation does not measure any relation between the flow velocity and the drag of the cylinder. Moreover, the Pearson correlation coefficient does not provide any kind of goal-oriented measure, mainly because it is linear in contrast to the transfer entropy which considers nonlinearities.

A quantitative measure of the error on the cylinder drag is provided in Fig. 7a. The error on the functional of interest $J({\textbf{u}}_{N})$ w.r.t. the reference solution $J({\textbf{u}}_{ref})$ is computed as $\delta J=|J({\textbf{u}}_{ref})-J({\textbf{u}}_{N})|$, similarly to Ref.^42^. The CSEI rapidly drops below $10^{-4}$ with only $N_{el}=600$ elements. This occurs because the refinement is aimed to improve the measurement of the functional, via the transfer entropy weighting. Afterwards, a plateau is reached as the large error in the wake drives the meshing. Differently, the SEI starts to increase the resolution in the wake and only after a few rounds even the front and near wake parts of the cylinder are solved accurately. In this way, a larger number of elements is required to have the same accuracy in the measurement of the drag.

## Discussion & conclusions

In this study, we begin discussing the transfer entropy (TE) estimation for two time series, whose samples are generated from a low-order vector auto-regressive (VAR) model. The importance of autoregressive models in constructing smooth archetypes for the (auto-) correlation function of turbulence time series was first pointed out by Oliver et al.^53^. This is required for an accurate estimation of uncertainties in turbulence first-order statistical moments. The main finding of our experiment is reported in Fig. 2b: the causality time scale is determined from the order of the VAR rather than the integral time scale of the time series which has been found relevant for uncertainty estimation in turbulence statistics^21,26^. Thus, the order of the VAR for modelling high-order statistics could be obtained from one of these time scales, with the integral one representing a conservative choice and without the need for any mathematical criteria for the order selection.

In turbulent flows, the transfer entropy shows a high potential to estimate the causal relationship between different flow variables and position in the flow. The advantage of the proposed approach is that the analysis can be performed either as a post-processing step based on the stored time series or, potentially, during the runtime as an in-situ analysis. To this end, we used the IDTxl Python toolkit^54^ applied to turbulent channel flow. We compare standard statistical quantities, e.g. the cross-covariance function, with the transfer entropy. The connection with the cross-covariance between autocorrelated time series is analysed in detail: when the cross-covariance reaches its maximum, the TE levels out (Fig. 5). Observing the relationship between these two statistical metrics is crucial since it enables the usage of TE as an alternative to cross-covariance/correlation, with the striking advantages of encapsulating a shift in the history effect and not suffering from significant spurious oscillations for very high lags. Furthermore, the TE analysis between wall friction and the streamwise velocity signal at different wall-normal distances proves the importance of the direction of information. The wall friction velocity $u_{\tau }$ influences *u* from the wall up to the upper limit of the buffer layer ($y^+ \approx 50$), but vice-versa only the immediate near-wall region ($y^+ \le 3$) *u* has a significant influence on $u_{\tau }$. This is a crucial outcome for wall modelling that aims to estimate accurate wall shear stress using flow data farther from the wall. In the standard wall modelling, the data from the logarithmic layer is commonly imported to a wall model. However, the results of our study show that the TE from the streamwise velocity to the wall shear stress varies with the wall distance. Thus, the magnitude of the TE can be used as an indicator of the sampling point to enhance the accuracy of the predicted wall shear stress. Note that the existing wall modelling approaches are mainly based on the correlations and still provide unsatisfactory results for more complex flow situations^55^.

As a final demonstration, a novel transfer entropy application is presented for data-driven error control in computational fluid dynamics. Our exploratory results indicate that TE can be used as a sensitivity measure, by computing the causality between different flow observables (state variables), thus linking them together in a directional sense. This information can then be used, in conjunction with local grid information, to drive a fully data-driven causality-based adaptive mesh refinement (AMR) strategy. This method may even be applicable in situations where other linear methods (such as adjoint sensitivity analysis) fail due to the exponential sensitivity to the initial conditions of chaotic dynamical systems. Furthermore, no additional equations need to be solved, leading to implementational and computational efficiency.

In conclusion, these initial yet significant results indicate the potential of transfer entropy in turbulent dynamical systems. They highlight a number of areas where directional causality information can be useful, if not critical. For the fully turbulent wall-bounded flows, we see an application of TE in the development of both data-driven error-controlling schemes and wall models where, during the training phase, it is necessary to separate causality-related relations from pure correlations. This is particularly relevant given the comparably low causality from the outer parts to the near-wall region of the flow discussed in Sect. "The turbulent channel flow", and the relation between cross-covariance (or similarly cross-correlation) and TE. Further studies, in particular at higher Reynolds numbers with more extensive scale separation and at varying flow conditions including pressure gradients, will certainly be enlightening as to understand the interactions between the various layers in the turbulent flow. Similar extensions of the TE concept can be thought of for the understanding of low-order models of more complex turbulent flows, e.g. when analysing cause-effect relations of large-scale instabilities. The potential of causal sensitivity analysis, in particular, related to adaptive meshing, is extremely interesting from a computational point of view. Since classical adjoint-based methods fail due to the exponential instabilities occurring at unsteady turbulent flows (or are impractically expensive^56^), the TE may provide at least an approximate way of quantifying the sensitivities which then, coupled with a suitable local error indicator, will give useful information for controlling the accuracy the QoI. Also, in this case, further studies, in particular for 3D turbulent flows will be considered in future.

The main drawbacks of an accurate estimation of TE are the computational cost, as discussed in Sect. "The turbulent channel flow", and the choice of the relevant hyperparameters. Through the extended application of the TE, we will certainly gain more experience and guidelines on choosing appropriate settings.

## Methods

### Causality as a measure

The causality definition has been long debated. Albeit the idea of cause-effect is well-established in our minds, its quantitative definition remains unclear. Here, we do not aim to discuss all possible options, rather we describe the metric adopted in the present work.

To this end, let us first recall some basic information theory concepts since the notion of information differs from our common meaning. Indeed, we deal with discrete random variables *Y*, which take values equal to *y* in a discrete (finite) set and have a probability density function (PDF) $p(y)=Pr(Y=y)$. Considering a given event $Y=y$, the information of *Y* is defined as:

5 $$\begin{aligned} I(y) = - \text {log}[p(y)] \,, \end{aligned}$$

where the logarithm base is arbitrarily chosen. On the one hand, the natural base *e* (the Euler’s number) expresses *I* in units of nats, where nat is the amount of information gained by observing an event of probability 1/*e*. On the other hand, base 2 expresses the information in bits. As the reader might be more familiar with the bits rather than nats, we look at an example of the information contained in the event *Y*(*heads*) for a fair coin-tossing $Y \in \{heads,tails\}$, where $p(heads)=p(tails)=0.5$. From now on, we abbreviate *heads* and *tails* as $h_d$ and $t_l$ respectively. Observe that we can always modify the information units by changing the base of logarithms. In base 2, we thus obtain information for $Y(h_d)$ equal to $I(Y=h_d) = 1$ bit, i.e. to communicate the result of our coin-tossing we just need 1 bit as this is a binary outcome.

In this context, Shannon^3^ generalised the thermodynamic entropy concept^4^ to an arbitrary variable. In this way, the Shannon entropy expresses the estimated mean value of *I*(*y*):

6 $$\begin{aligned} H(Y) = \overline{I(Y)}= \sum _y -p(y) \text {log}{[p(y)]} \ge 0. \end{aligned}$$

This defines the average number of bits, or nats, to express *n* independent draws of a discrete variable *Y* which follows a distribution *p*(*y*):

7 $$\begin{aligned} H(Y) = -p(h_d)\text {log}[p(h_d)] -p(t_l)\text {log}[p(t_l)]\,. \end{aligned}$$

For the fair coin example, the Shannon entropy results in $H(Y)=1$. Considering now a biased coin-tossing, where we can only get heads with the probabilities $p(h_d)=1$ and hence $p(t_l)=0$: The Shannon entropy returns $H(Y)=0$. This straightforward example demonstrates how the Shannon entropy can measure the uncertainty of the state. Indeed, when all the outcomes are equiprobable, *H* is maximum while *H* becomes zero when the process is completely deterministic with no uncertainty.

The last piece of the puzzle to compute Schreiber’s causality is the Kullback entropy *K*^57^. For an event *Y*, *K* measures the divergence between two probability distributions which are both related to the discrete state *y*:

8 $$\begin{aligned} K(Y) = \sum _y p(y) \text {log}{\frac{p(y)}{q(y)}}. \end{aligned}$$

When the Kullback entropy is used for a conditional probability, it is often called Kullback–Leiber divergence:

9 $$\begin{aligned} K(Y|X) = \sum _{y,x} p(y,x) \text {log}{\frac{p(y|x)}{q(y|x)}}\,, \end{aligned}$$

as it provides a null divergence when the event *Y* is fully independent of *X*.

With this in mind, we now move to the meaning of causation. In 1956, Wiener^9^ introduced the following idea: *“A signal X is said to cause Y when the future of signal Y is better predicted by adding knowledge from the past and present of signal X, rather than by using present/past of Y alone”*. Several years later, in 2000, Schreiber^2^ proposed a rigorous derivation, starting from the generalised Markov condition:

10 $$\begin{aligned} p(y_{t}|{\textbf{y}_{\textbf{t}}^{\textbf{n}}},{\textbf{x}_{\textbf{t}}^{\textbf{m}}})=p(y_{t}|{\textbf{y}_{\textbf{t}}^{\textbf{n}}}) \,, \end{aligned}$$

where *m* and *n* are the orders of Markov process for the source *x* and target *y*:

11 $$\begin{aligned} {{\textbf{y}}_{\textbf{t}}^{\textbf{n}}}= & {} (y_{t-1},...,y_{t-n}), \end{aligned}$$

12 $$\begin{aligned} {{\textbf{x}}_{\textbf{t}}^{\textbf{m}}}= & {} (x_{t-1},...,x_{t-m}), \end{aligned}$$

with $m,n \ge 1$. In the preceding sections, the Markovian orders are associated with $\tau$, while $\tau _{min}$ represents a time shift in the past. This shift allows us to exclude the influence of a specific interval on the current state, see Fig. 2a. The relation (10) is fully satisfied when the *y*-dynamics is independent of the present and past of *x*. When it is not, Schreiber^2^ uses the Kullback–Leiber entropy to measure the divergence of relation (10) which results in:

13 $$\begin{aligned} TE_{X \rightarrow Y}= \sum _{y_{t},{\textbf{y}_{\textbf{t}}^{\textbf{n}}},{\textbf{x}_{\textbf{t}}^{\textbf{m}}}} p(y_{t},{\textbf{y}_{\textbf{t}}^{\textbf{n}}},{\textbf{x}_{\textbf{t}}^{\textbf{m}}}) \text {log}\frac{p(y_{t}|{\textbf{y}_{\textbf{t}}^{\textbf{n}}},{\textbf{x}_{\textbf{t}}^{\textbf{m}}})}{p(y_{t}|{\textbf{y}_{\textbf{t}}^{\textbf{n}}})} \,. \end{aligned}$$

Eventually, expression (13) can be re-written as sum of four Shannon entropies:

14 $$\begin{aligned} TE_{X \rightarrow Y}= & {} H({\textbf{y}}_t^n,{\textbf{x}}_t^m)-H(y_t,{\textbf{y}}_t^n,{\textbf{x}}_t^m) \nonumber \\{} & {} +H(y_t,{\textbf{y}}_t^n)-H({\textbf{y}}_t^n). \end{aligned}$$

From the current formulation, it follows that $TE_{X \rightarrow Y}$ varies between 0 (no causation) and 1 (*Y* is only caused by *X*)^2^.

### Transfer entropy calculation

Concretely, the transfer entropy estimation concerns the computation of the different joint and marginal probability distributions introduced in Eq. (14). In the literature, we identify two macro-categories for probability distribution estimation: parametric and non-parametric approaches^58^. In the parametric approach, an estimator is designed based on the assumption of a certain model for the PDF, the choice of which is often difficult to justify. Furthermore, we need to find the parameters that optimally fit the sample probability densities to our assumed distribution.

We, instead, use a non-parametric approach, namely the nearest-neighbour estimator^59^, which avoids arbitrariness or biases arising from binning^60,61^. The nearest-neighbour technique is also known as $\kappa$NN or $\kappa$-NN, since it considers the $\kappa$-th nearest neighbour, and guarantees an adaptive resolution scaling the chosen distance with the density. Kozachenko and Leonenko (KL)^62^ introduced an asymptotically unbiased estimator based on $\kappa$-NN statistics assuming a constant distribution of the random vectors within the neighbourhood. In the final entropy estimation, the following approach gives a bias depending on the dimensionality size. Kraskov et al.^63^ first observed that the same formulation holds for varying $\kappa$ values. As a fixed $\kappa$ is not required, the bias limitations that occur with individual KL estimates are overcome and an exact estimator for independent variables is obtained. In the current study, we rely on such an approach using the IDTxl Python toolkit^54^, widely employed in the IT community^64–67^.

Albeit the $\kappa$-NN kernel estimator is classified as a non-parametric approach, since no model for the PDF is assumed, some parameters need to be chosen, starting from the mass of the nearest-neighbours $\kappa$. Following Kraskov et al.^63^, we employ a mass equal to 4. Two other hyperparameters which might severely affect the TE evaluation are the following: the embedding delays $\tau$, i.e. the Markovian orders *m* and *n* for the target and the source in (11) and (12), respectively, and the Theiler correction window which excludes the auto-correlation effects from the estimation^63^. The Theiler correction is usually set to 1 and the Markovian order *m* for the target is also taken to be 1 since we are interested in the past/present source causation on the current target. These settings are used in the results presented above (see also the Appendix). Additionally, the numerical approach necessitates specifying the dimensionality of the time series, including the number of samples (denoted as $n_t$) and the number of permutations (denoted as $n_p$) used for statistical significance. The number of permutations corresponds to the times the samples are shuffled to generate surrogate data, from which TE is subsequently re-estimated.

### Direct numerical simulation using spectral-element method

The direct numerical simulations (DNS) have been carried out with the spectral-element code Nek5000^41^. The code uses the spectral element method (SEM)^68^ which combines local spectral accuracy, i.e. nearly exponential error convergence, and geometrical flexibility of finite element methods. With the $P_N-P_{N-2}$ formulation, the functional spaces for the velocity and pressure are spanned by the Lagrange interpolants integrated over Gauss–Lobatto–Legendre points (GLL) and Gauss–Legendre (GL) points, respectively. In the simulations discussed, the polynomial order in each element is $N=7$. Regarding the time integration, we use a third-order implicit backward differentiation (BDF) with an extrapolation scheme of order three for the convective term.

The DNS of the incompressible turbulent flow between two parallel planes at $Re_{\tau }=300$ is performed^69^. Periodic boundary conditions are applied in the streamwise, *x*, and spanwise, *z*, directions. No-slip/no-penetration boundary conditions are applied at the wall. The computational domain sizes are $L_x=2\pi h$, $L_z=\pi h$ and $L_y=2 h$, where *h* is the channel half-height. The flow is driven by a fixed mass flux which translates to a constant bulk velocity for constant-density flows. The numerical setup is shown in Fig. 3.

For the AMR application, the two-dimensional flow around a circular cylinder is simulated at $Re_D=200$, where the Reynolds number is based on the incoming velocity, the cylinder diameter (*D*) and the kinematic viscosity $\nu$. The computational domain is a box expanded in the streamwise and spanwise directions as $(-10D,20D)$ and $(-10D,10D)$, respectively. The left boundary is an inlet with constant non-dimensional velocity ${\textbf{u}}(\textbf{x}_{\textbf{0}},t)=(1,0,0)$. The side boundaries are periodic, and no-slip and impermeability conditions are imposed at the cylinder’s surface. After discarding the initial transient, we collect the time series for the spectral error indicator and the velocity magnitude (at each element’s centre) for at least 10 vortex shedding periods.

### Adaptive mesh refinement

Adaptive mesh refinement (AMR) is a technique for error-driven meshing with the improvement of the overall numerical accuracy based on an error indicator. Thus, higher Reynolds numbers and more complicated geometries, with a significant reduction of the computational costs are allowed^40^.

Our group has implemented the AMR in Nek5000 keeping the good properties of the native code, e.g. the high scalability, fast convergence and favourable numerical properties^47^. The two main ingredients of the AMR algorithm are the error indicator (or estimator) and the refinement method. Concerning the latter, among the different possibilities available in the literature, the *h*-refinement scheme is used. It consists of splitting isotropically the elements and refining and coarsening the mesh locally. The hanging nodes at the non-conforming interfaces are treated via the parent-to-children interpolating operator^70^. To handle the grid hierarchy of the mesh and to perform parallel partitioning, we rely on the external libraries p4est^71^ and parMetis^72^, and parRSB, respectively. Further details for the interested reader can be found in Offermans^47^. When it comes to measuring the error, we use the spectral error indicator (SEI) which was introduced first by Mavriplis^46^. The SEI is a local measure of the truncation and quadrature errors combined for the solution fields. To understand the SEI, we can look at a simple problem where *u* is the exact solution to a system of one-dimensional partial differential equations and $u_N$ is a corresponding approximate spectral-element solution with polynomials of order *N*. We expand *u*(*x*) on a reference element in terms of the Legendre polynomials as,

15 $$\begin{aligned} u(x) = \sum _{p=0}^{\infty } {\hat{u}}_p L_p(x) \,, \end{aligned}$$

where ${\hat{u}}_p$ are the associated spectral coefficients and $L_p(x)$ is the Legendre polynomial of order *p*. The estimated error $\varepsilon = \Vert u-u_N \Vert _{L^2}$ results in:

16 $$\begin{aligned} \varepsilon = \Biggl ( \int _{N}^{\infty } \frac{{\hat{u}}(p)^2}{\frac{2p+1}{2}} dp + \frac{{\hat{u}}^2_N}{\frac{2N+1}{2}} \Biggr )^{\frac{1}{2}}\,, \end{aligned}$$

where we assume an exponential decay for the spectral coefficients of the form ${\hat{u}}(p) \approx c \hspace{0.5mm} \text {exp}(-\sigma p)$. The parameters *c* and $\sigma$ are obtained via interpolating in a linear least-squares sense the $\text {log}({\hat{u}}_p)$ for $p\le N$. In a three-dimensional problem, the maximum error among each component is considered, providing a single spectral indication per element. As we do not aim to track instantaneous features in time, but rather converge to a statistically stationary mesh, we perform a time-average of $\varepsilon$ for a given interval *T*. In this study, we use the transfer entropy as a weighting function for the SEI, to directly incorporate the causality in the error-reduction mesh adaptation scheme (Supplementary Information).

### Supplementary Information

Supplementary Information.

## Data Availability

All data needed to evaluate the conclusions are present in the paper. Additional data related to this work are available upon reasonable request to the corresponding authors.
