# Supplementary material for: On the potential of transfer entropy in turbulent dynamical systems
Source: Sci Rep. 2023 Dec 15;13:22344. doi: 10.1038/s41598-023-49747-1 (PMC10724263; doi:10.1038/s41598-023-49747-1)
Supplement: Supplementary file 1 — Supplementary Information. [file 41598_2023_49747_MOESM1_ESM.pdf]

On the potential of transfer entropy in turbulent dynamical systems

Daniele Massaro*,[1] Saleh Rezaeiravesh,[1,2] and Philipp Schlatter[1,3]

[1]*SimEx/FLOW, KTH Engineering Mechanics,*
Royal Institute of Technology, Stockholm, Sweden
[2]*Department of Fluids and Environment/MACE,*
The University of Manchester, Manchester, M139PL, UK
[3]*Institute of Fluid Mechanics (LSTM),*
Friedrich–Alexander Universität (FAU), Erlangen-Nürnberg, Germany

(Dated: October 28, 2023, (*dmassaro@kth.se*))

		$T \sim 40$	$T \sim 160$	$T \sim 320$	$T \sim 600$	$T \sim 740$	$T \sim 836$
$y^+ = 0.55$	$(s = u_\tau)$	0.287	0.426	0.527	0.636	0.695	0.696
	$(s = u)$	0.271	0.538	0.538	0.651	0.708	0.710
$y^+ = 18$	$(s = u_\tau)$	0.219	0.272	0.299	0.329	0.338	0.338
	$(s = u)$	0.036	0.040	0.047	0.041	0.041	0.0.041
$y^+ = 47$	$(s = u_\tau)$	0.270	0.307	0.332	0.352	0.356	0.356
	$(s = u)$	0.043	0.047	0.047	0.047	0.050	0.050
$y^+ = 81$	$(s = u_\tau)$	0.264	0.307	0.332	0.352	0.368	0.368
	$(s = u)$	0.037	0.047	0.047	0.047	0.047	0.047

TABLE I. The transfer entropy is computed between the time series of the friction and streamwise velocities at different y^+ locations. At each y^+, we consider each of the friction velocity and streamwise velocity as the source. Time series of different sample size T (normalised by U_b/h) are considered.

APPENDIX

One of the primary limitations of the transfer entropy as a causality metric is the statistical convergence of its estimations. Previous studies indicate that the Shannon TE calculation is not significantly affected by the sample size [23]. However, this could not be (always) the case in a highly nonlinear dynamical system like turbulent flows. The literature lacks comprehensive studies where the effect of the time series size is assessed. Thus, we believe that reporting the convergence of our measurements is crucial. In the turbulent channel flow at the moderate Reynolds number $Re_\tau = 300$, we sample with a constant time-step size $\Delta t = 0.008 U_b/h$ (U_b is the bulk velocity and h is the channel half height). Velocity time series are collected for approximately 800 convective time units, based on the bulk velocity U_b and channel half-height h (corresponding to approximately ≈ 40 ETT). The estimated TEs for different sample sizes are reported in table I.

First, we observe different rates of convergence at various y^+. Notably, as one moves farther from the wall, the TE converges more rapidly. Second, when the causal relation is stronger ($\gtrsim 0.5$), meaning that the majority of information about the target originates from the target itself rather than from various other sources, the convergence rate is slower. Overall, across the range of the wall-normal locations considered here, the sample size seems

sufficient to ensure a converged Shannon TE estimation. It is worth noting that the spatial averaging in both the spanwise and streamwise directions may have further enhanced the convergence of transfer entropy by reducing the space dimensionality.

---

[1] G. Galilei, *Il saggiatore* (1624).

[2] T. Schreiber, Measuring information transfer, Phys. Rev. Let. **85**, 461 (2000).

[3] C. E. Shannon, A mathematical theory of communication, Bell Syst. Tech. **27**, 379 (1948).

[4] L. Boltzmann, On the relationship between the second fundamental theorem of the mechanical theory of heat and probability calculations regarding the conditions for thermal equilibrium, Wiss. Abhandlungen **2**, 164 (1909).

[5] A. S. Eddington, *The Nature of the Physical World* (Cambridge University Press, 1929).

[6] S. Harvey and A. Rex, *Maxwell's Demon: Entropy, Information, Computing* (Princeton Series in Physics, 1990).

[7] X. S. Liang, Information flow within stochastic dynamical systems, Phys. Rev. E **78**, 031113 (2008).

[8] A. Lozano-Durán and G. Arranz, Information-theoretic formulation of dynamical systems: causality, modeling, and control, Phys. Rev. Res. **4**, 023195 (2022).

[9] N. Wiener, *The Theory of Prediction, Modern Mathematics for Engineers* (McGraw-Hill, New York, 1956).

[10] T. Bossomaier, L. Barnett, M. Harré, and J. T. Lizier, *An Introduction to Transfer Entropy. Information Flow in Complex Systems* (Springer, 2016).

[11] F. A. Razak and H. J. Jensen, Quantifying 'causality' in complex systems: understanding transfer entropy, PLoS One **9**, 6 (2014).

[12] R. G. James, N. Barnett, and J. P. Crutchfield, Information flows? A critique of transfer entropies, Phys. Rev. Lett. , 238701 (2016).

[13] M. Beneitez, *Nonlinear dynamics in transitional wall-bounded flows*, Ph.D. thesis, KTH Royal Institute of Technology, Stockholm, Sweden (2021).

[14] An animated map of global weather conditions:, NASA, https://earth.nullschool.net/ (2023).

[15] B. Ünal, Causality analysis for COVID-19 among countries using effective transfer entropy, Entropy **24**, 1115 (2022).

[16] T. Imaizumi, N. Umeki, R. Yoshizawa, T. Obuchi, Y. Sako, and Y. Kabashima, Assessing transfer entropy from biochemical data, Phys. Rev. E **105**, 034403 (2022).

[17] E. N. Lorenz, Deterministic nonperiodic flow, J. Atm. Sci. **20**, 130–141 (1963).

[18] C. Sparrow, *The Lorenz Equations. Bifurcations, Chaos, and Strange Attractors* (Springer–Verlag, 1982).

[19] W. W. S. Wei, *Time Series Analysis: Univariate and Multivariate Methods* (Pearson, 1190).

[20] M. Min, M. Brazell, A. Tomboulides, M. Churchfield, P. Fischer, and M. Sprague, Towards exascale for wind energy simulations (2022), arXiv:2210.00904.

[21] S. Rezaeiravesh, D. Xavier, R. Vinuesa, J. Yao, F. Hussain, and P. Schlatter, Estimating uncertainty of low- and high-order turbulence statistics in wall turbulence, in *Twelfth International Symposium on Turbulence and Shear Flow Phenomena (TSFP12)* (2022).

[22] R. H. Shumway and D. S. Stoffer, *Time Series Analysis and Its Applications* (Springer Cham, 2017).

[23] P. Wollstadt, M. Martínez-Zarzuela, R. Vicente, F. Díaz-Pernas, and M. Wibral, Efficient transfer entropy analysis of non-stationary neural time series, PLoS ONE **9**, 7 (2014).

[24] C. Amornbunchornvej, E. Zheleva, and T. Berger-Wolf, Variable-lag granger causality and transfer entropy for time series analysis, ACM Trans. Know. Disc. Data **15**, 1 (2021).

[25] K. Park, *Fundamentals of Probability and Stochastic Processes with Applications to Communications* (Springer, 2008).

[26] D. Xavier, S. Rezaeiravesh, R. Vinuesa, and P. Schlatter, Autoregressive model-based estimator for quantifying time-averaging uncertainties in turbulent flows, To be submitted (2023).

[27] S. B. Pope, *Turbulent Flows* (Cambridge University Press, 2000).

[28] G. Tissot, A. Lozano-Durán, J. Jiménez, L. Cordier, and B. R. Noack, Granger causality in wall-bounded turbulence, J. Phys.: Conf. Ser. **506** (2014).

[29] C. Granero-Belinchón, S. G. Roux, and N. B. Garnier, Scaling of information in turbulence, Europhys. Lett. **115**, 5 (2016).

[30] C. Granero-Belinchón, S. G. Roux, and N. B. Garnier, Kullback-Leibler divergence measure of intermittency: application to turbulence, Phys. Rev. E **97**, 013107 (2018).

[31] C. Granero-Belinchón, S. G. Roux, and N. B. Garnier, Quantifying non-stationarity with information theory, Entropy **23**, 1609 (2021).

[32] A. Lozano-Durán, H. J. Bae, and M. P. Encinar, Causality of energy-containing eddies in wall

turbulence, J. Fluid Mech. **882**, A2 (2019).

[33] W. Wang, X. Chu, A. Lozano-Durán, R. Helmig, and B. Weigand, Information transfer between turbulent boundary layers and porous media, J. Fluid Mech. **920**, A21 (2021).

[34] J. Jimènez, Coherent structures in wall-bounded turbulence, J. Fluid Mech. **842**, P1 (2018).

[35] M. Quadrio and P. Ricco, Critical assessment of turbulent drag reduction through spanwise wall oscillation, J. Fluid Mech. **521**, 251–271 (2009).

[36] R. J. Adrian, Hairpin vortex organization in wall turbulence, Phys. Fluids **19**, 041301 (2007).

[37] J. A. Sillero, J. Jimènez, and R. Moser, Two-point statistics for turbulent boundary layers and channels at reynolds numbers up to $\delta^+ \approx 2000$, Phys. Fluids **26**, 105109 (2014).

[38] J. Slotnick, A. Khodadoust, J. Alonso, D. Darmofal, W. Gropp, E. Lurie, and C. Mavriplis, CFD Vision 2030 Study: A Path to Revolutionary Computational Aeroscience, Report by National Aeronautics and Space Administration (2014).

[39] P. Fischer, J. Kruse, J. Mullen, H. Tufo, J. Lottes, and S. Kerkemeier, Nek5000: open source spectral element CFD solver, https://nek5000.mcs.anl.gov/ (2008).

[40] N. Offermans, A. Peplinski, O. Marin, and P. Schlatter, Adaptive mesh refinement for steady flows in Nek5000, Comp. Fluids **197**, 104352 (2020).

[41] N. Offermans, D. Massaro, A. Peplinski, and P. Schlatter, Error-driven adaptive mesh refinement for unsteady turbulent flows in spectral-element simulations, Comp. Fluids **251**, 105736 (2023).

[42] D. Massaro, A. Peplinski, and P. Schlatter, Interface discontinuities in spectral-element simulations with adaptive mesh refinement, in *Spectral and High Order Methods for Partial Differential Equations ICOSAHOM 2020+1. Lecture Notes in Computational Science and Engineering* (Springer International Publishing, 2023) pp. 375–386.

[43] D. Massaro, V. Lupi, A. Peplinski, and P. Schlatter, Global stability of 180°-bend pipe flow with mesh adaptivity, Phys. Rev. Fluids (in press) (2023).

[44] C. Mavriplis, *Nonconforming Discretizations and a Posteriori Error Estimators for Adaptive Spectral Element Techniques*, Ph.D. thesis, Massachusetts Institute of Technology, Massachusetts, USA (1989).

[45] N. Offermans, *Aspects of adaptive mesh refinement in the spectral element method*, Ph.D. thesis, KTH Royal Institute of Technology, Stockholm, Sweden (2019).

[46] D. Massaro, A. Peplinski, and P. Schlatter, Direct numerical simulation of turbulent flow

around 3D stepped cylinder with adaptive mesh refinement, in *Twelfth International Symposium on Turbulence and Shear Flow Phenomena (TSFP12)* (2022).

[47] D. Massaro, A. Peplinski, and P. Schlatter, The flow around a stepped cylinder with turbulent wake and stable shear layer, (submitted) (2023).

[48] D. Massaro, A. Peplinski, and P. Schlatter, Coherent structures in the turbulent stepped cylinder flow at $Re_D = 5000$, Int. J. Heat and Fluid Flow **102**, 109144 (2023).

[49] N. Chandramoorthy and Q. Wang, Sensitivity computation of statistically stationary quantities in turbulent flows, AIAA Aviation 2019 Forum , 3426 (2019).

[50] W. Bangerth and R. Rannacher, *Adaptive Finite Element Methods for Differential Equations* (Birkhäuser, Basel, 2002).

[51] T. A. Oliver, N. Malaya, R. Ulerich, and R. D. Moser, Estimating uncertainties in statistics computed from direct numerical simulation, Phys. Fluids **26**, 035101 (2014).

[52] P. Wollstadt, J. T. Lizier, R. Vicente, M. M.-Z. C. Finn, P. Mediano, L. Novelli, and M. Wibral, Idtxl: the information dynamics toolkit xl. a python package for the efficient analysis of multivariate information dynamics in networks, J. Op. Sour. Soft. **23**, 34 (2019).

[53] J. Larsson, S. Kaway, J. Bodarat, and I. Bermejo-Moreno, Large eddy simulation with modeled wall-stress: recent progress and future directions, Mech. Eng. Rev. **3**, 15 (2016).

[54] Q. Wang, Convergence of the least squares shadowing method for computing derivative of ergodic averages, SIAM Journal on Numerical Analysis **52**, 156 (2014).

[55] S. Kullback, *Information Theory and Statistics* (Wiley, New York, 1959).

[56] K. Hlavackova-Schindler, M. Palus, M. Vejmelka, and J. Bhattacharya, Causality detection based on information-theoretic approaches in time series analysis, Phys. Rep. **441**, 1 (2007).

[57] S. B. W., *Density estimation for statistics and data analysis* (CRC press, 1986).

[58] J. Victor, Binless strategies for estimation of information from neural data, Phys. Rev. E **66**, 051903 (2002).

[59] A. Kaiser and T.Schreiber, Information transfer in continuous processes, Physica **110**, 43 (2002).

[60] L. Kozachenko and N. Leonenko, Sample estimate of the entropy of a random vector, Prob. Pered. Info. **23** (1987).

[61] A. Kraskov, H. Stogbauer, and P. Grassberger, Estimating mutual information, Phys. Rev. E **69**, 066138 (2004).

[62] M. R. Gonzalez, T. Basse, D. Saft, and F. Kunze, Leading indicators for US house prices: new evidence and implications for EU financial risk managers. European Financial Management, Eur. Fin. Manag. **28**, 3 (2021).

[63] P. Wollstadt, M. Hasenjäger, and C. B. Wiebel-Herboth, Quantifying the predictability of visual scanpaths using active information storage, Entropy **23**, 167 (2021).

[64] M. S. Harre, Information theory for agents in artificial intelligence, psychology, and economics, Entropy **23**, 310 (2021).

[65] L. Novelli, F. M. Atay, J. Jost, and J. T. Lizier, Deriving pairwise transfer entropy from network structure and motifs, Proc. Royal Soc. A **476**, 2236 (2020).

[66] A. Patera, A spectral element method for fluid dynamics: laminar flow in a channel expansion, J. Comput. Physics **54**, 468 (1984).

[67] D. Massaro, A. Peplinski, R. Stanly, S. Mirzareza, V. Lupi, T. Mukha, and P. Schlatter, A comprehensive framework to enhance numerical simulations in the spectral-element code Nek5000, (submitted) (2023).

[68] G. W. Kruse, *Parallel Nonconforming Spectral Element Solution of the Incompressible Navier–Stokes Equations in Three Dimensions*, Ph.D. thesis, Brown University, Rhode Island, USA (1997).

[69] C. Burstedde, L. C. Wilcox, and O. Ghattas, p4est: scalable algorithms for parallel adaptive mesh refinement on forests and octrees, SIAM J. Sci. Comput. **33**, 1103–1133 (2011).

[70] G. Karypis, K. Schloegel, and V. Kumar, *ParMeTiS: Parallel graph partitioning and sparse matrix ordering library*, Tech. Rep. 97-060 (Comp. Sci. and Eng., 1997).

[71] D. Massaro, S. Rezaeiravesh, and P. Schlatter, Causality-based algorithms for adaptive mesh refinement in turbulent flow simulations, in *https://torroja.dmt.upm.es/causturb/* (Abstract, 2022).
